# β3 adrenergic agonism: A novel pathway which improves right ventricular‐pulmonary arterial hemodynamics in pulmonary arterial hypertension

**DOI:** 10.14814/phy2.15549

**Published:** 2023-01-03

**Authors:** Keyvan Karimi Galougahi, Yunjia Zhang, Vivian Kienzle, Chia‐Chi Liu, Lake‐Ee Quek, Sanjay Patel, Edmund Lau, Rachael L. Cordina, Gemma A. Figtree, David S. Celermajer

**Affiliations:** ^1^ Heart Research Institute Sydney Australia; ^2^ Royal Prince Alfred Hospital Sydney Australia; ^3^ Sydney Medical School Faculty of Medicine and Health University of Sydney Sydney Australia; ^4^ Kolling Institute for Medical Research Sydney Australia; ^5^ Charles Perkins Center University of Sydney Sydney Australia; ^6^ Department of Respiratory Medicine Royal Prince Alfred Hospital Sydney Australia; ^7^ Department of Cardiology Royal North Shore Hospital Sydney Australia

**Keywords:** endothelial nitric oxide synthase, pulmonary arterial hypertension, redox signaling, right ventricular hemodynamics, β3 adrenergic receptors

## Abstract

Efficacy of therapies that target the downstream nitric oxide (NO) pathway in pulmonary arterial hypertension (PAH) depends on the bioavailability of NO. Reduced NO level in PAH is secondary to “uncoupling” of endothelial nitric oxide synthase (eNOS). Stimulation of β3 adrenergic receptors (β3 ARs) may lead to the recoupling of NOS and therefore be beneficial in PAH. We aimed to examine the efficacy of β3 AR agonism as a novel pathway in experimental PAH. In hypoxia (5 weeks) and Sugen hypoxia (hypoxia for 5 weeks + SU5416 injection) models of PAH, we examined the effects of the selective β3 AR agonist CL316243. We measured echocardiographic indices and invasive right ventricular (RV)–pulmonary arterial (PA) hemodynamics and compared CL316243 with riociguat and sildenafil. We assessed treatment effects on RV–PA remodeling, oxidative stress, and eNOS glutathionylation, an oxidative modification that uncouples eNOS. Compared with normoxic mice, RV systolic pressure was increased in the control hypoxic mice (*p* < 0.0001) and Sugen hypoxic mice (*p* < 0.0001). CL316243 reduced RV systolic pressure, to a similar degree to riociguat and sildenafil, in both hypoxia (*p* < 0.0001) and Sugen hypoxia models (*p* < 0.03). CL316243 reversed pulmonary vascular remodeling, decreased RV afterload, improved RV–PA coupling efficiency and reduced RV stiffness, hypertrophy, and fibrosis. Although all treatments decreased oxidative stress, CL316243 significantly reduced eNOS glutathionylation. β3 AR stimulation improved RV hemodynamics and led to beneficial RV–PA remodeling in experimental models of PAH. β3 AR agonists may be effective therapies in PAH.

## Graphical abstract

In pulmonary arterial hypertension, endothelial nitric oxide synthase is “uncoupled” via glutathionylation, leading to decreased generation of NO and increased $O_{2}^{\cdot -}$. These changes lead to impaired NO‐dependent signaling in smooth muscle cells, resulting in increased proliferation and vasoconstriction, and increased pulmonary arterial (PA) pressure. Consequently, the right ventricular (RV)–PA coupling efficiency is impaired, and RV becomes hypertrophied and fibrosed. Stimulation of β3 adrenergic receptors by CL316243 leads to a decrease in glutathionylation‐mediated uncoupling of eNOS, decreased $O_{2}^{\cdot -}$, and increased NO‐dependent signaling, which lead to decreased proliferation and increased vasodilation, hence a decrease in PA pressure. The reduction in RV afterload results in improved RV–PA coupling efficiency and reverse remodeling of RV (reduced hypertrophy and fibrosis).

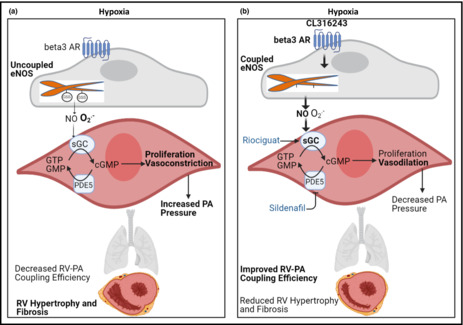

## 1 INTRODUCTION

Pulmonary arterial hypertension (PAH) is a life‐threatening disease that is characterized by increased pulmonary vascular resistance due to progressive vascular remodeling, which can ultimately lead to right heart failure and death (Humbert et al., 2019). Current therapies include prostanoids, endothelin‐receptor antagonists (e.g., bosentan), and agents that activate nitric oxide (NO) signaling (i.e., phosphodiesterase type 5 [PDE5] inhibitors such as sildenafil and soluble guanylyl cyclase [sGC] activators such as riociguat; Simonneau et al., 2019). Mortality, however, remains high despite treatment, and there is a considerable unmet need in the management of PAH (Humbert et al., 2019).

In the canonical NO‐sGC‐cyclic guanosine monophosphate (cGMP) signaling pathway, NO is synthesized in the pulmonary endothelial cells via the conversion of L‐arginine to NO and citrulline by NO synthase (NOS), with endothelial NOS (eNOS)‐mediated biosynthesis being the major source of NO in the pulmonary circulation (Hansen et al., 2016). In PAH, the bioavailability of NO is diminished, primarily due to the altered function of eNOS, (i.e., “uncoupling” of eNOS) that leads to the generation of $O_{2}^{\cdot -}$ instead of NO (Chen et al., 2010). Thus, in addition to the approved therapies that enhance the sGC‐cGMP pathway, targeting the uncoupling of eNOS may be beneficial in the treatment of PAH (Hansen et al., 2016).

The β3 adrenergic receptor (β3 AR) belongs to the G protein‐coupled family of receptors and is expressed in ventricular myocytes (Michel et al., 2020) and vascular endothelial cells (Karimi Galougahi, Liu, et al., 2016). β3 AR colocalizes with eNOS in the caveolae‐enriched cellular membrane rafts, and its activation is directly coupled to sGC‐cGMP signaling (Michel et al., 2020). We have previously shown that β3 AR stimulation enhances NO bioavailability and reduces redox stress in the systemic circulation (Karimi Galougahi, Liu, et al., 2016). In the present study, we investigated the effects of β3 AR stimulation on pulmonary hemodynamics, right ventricular (RV)–pulmonary artery (PA) coupling efficiency, and compared the putative efficacy of β3 AR stimulation to guideline‐recommended agents that potentiate the downstream sGC‐cGMP pathway in two experimental models of PAH.

## 2 METHODS

All study protocols were approved by the Animal Ethics Committee of Sydney Local Health District (protocol number 2019/007A) and University of Sydney (protocol number 2019/1553), Sydney, Australia. The inclusion of human tissue samples in the study was approved by the Human Research Ethics Committee of the Northern Sydney Local Health District, Sydney, Australia.

### 2.1 Experimental models of PAH and treatments

**FIGURE 1:**
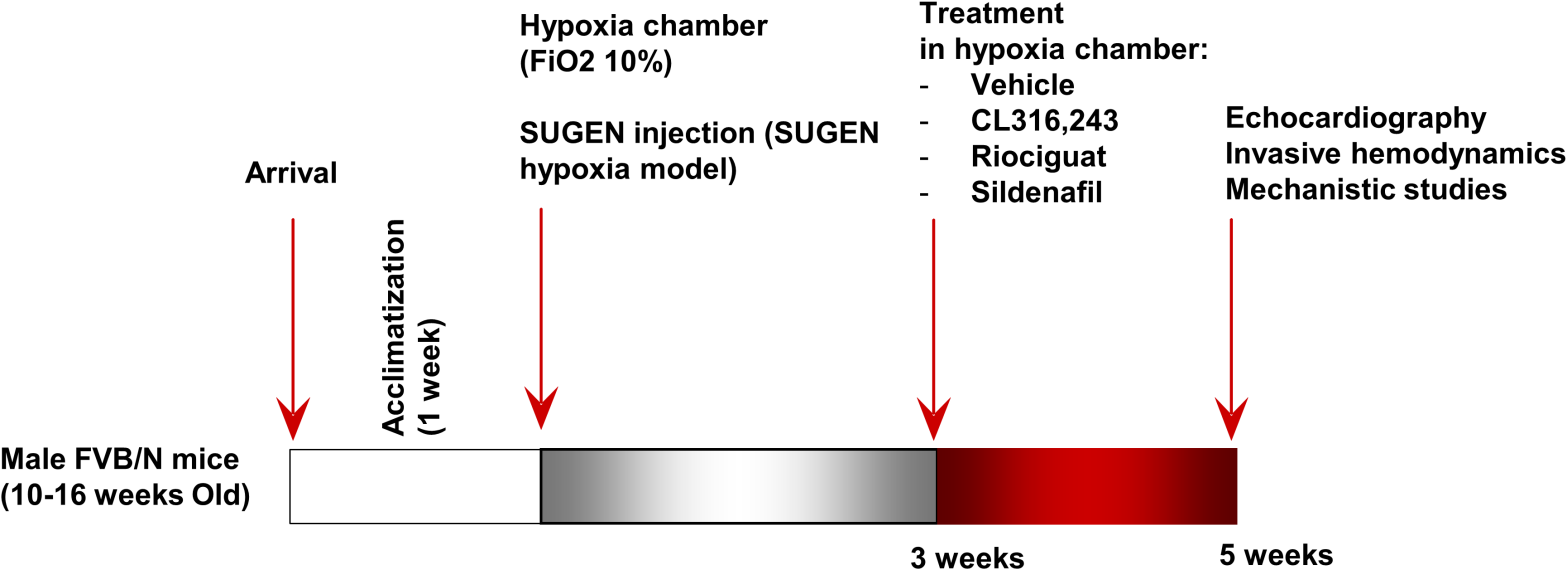
Study protocol. FiO_2_, fraction of inspired O_2_.

Using adult male FVB/N mice (Gomez‐Arroyo et al., 2012; 10–16 weeks old) with a body weight of 20–25 g (Australian BioResources), we established two models of PAH: (i) hypoxia model (Hx), where mice were exposed to chronic hypoxia (fraction of inspired O_2_ [FiO_2_] = 0.10) for 3 weeks in a hypoxia chamber (BioSpherics), (ii) Sugen hypoxia model (SUHx), where mice were exposed to chronic hypoxia (FiO_2_ = 0.10) for 3 weeks and received a weekly subcutaneous injection of the vascular endothelial growth factor receptor inhibitor SU5416 (20 mg/kg, Sigma‐Aldrich; total three injections, Figure 1; Lang et al., 2012). In both models, mice were housed in the hypoxia chamber for an additional 2 weeks during which they were treated by daily gavage with riociguat (MedChemExpress) suspended in 1% (w/v) dimethylcellulose at a dose of 10 mg/kg/day (*n* = 10/model; Lang et al., 2012), or sildenafil (MedChemExpress) dissolved in distilled water at a dose of 50 mg/kg/day (*n* = 10/model; Lang et al., 2012), or with the selective β3 AR agonist CL316243 (Sigma‐Aldrich) dissolved in saline via osmotic minipump (Alzet) at an infusion rate of 40 μg/kg/h (*n* = 10/model; Karimi Galougahi, Liu, et al., 2016). Mice receiving vehicle for 2 weeks (i.e., gavaged daily with a mixture of solvents in both models as well as receiving a saline injection in the Hx model or a mixture of saline and carboxymethylcellulose in the SUHx model) and exposed to FiO_2_ = 0.1 or the ambient air for 5 weeks were used as control groups (*n* = 20).

### 2.2 Echocardiography

Transthoracic echocardiography was performed in lightly sedated mice (isoflurane 1%) with a Vevo 2100 high‐resolution imaging system equipped with a 30 MHz transducer (VisualSonics). Pulmonary artery acceleration time (PAT), a noninvasive estimate of PA pressure (Beloiartsev et al., 2015), RV wall thickness, and left ventricular (LV) systolic and diastolic internal diameters and ejection fraction were measured (Beloiartsev et al., 2015). Analysis was performed on Vevo Lab software (VisualSonics) by an investigator blinded to treatment assignments.

### 2.3 Invasive hemodynamic measurements

Pressure–volume loops were acquired in intubated and ventilated mice at a respiratory rate of 120 breaths·min^−1^, a tidal volume of 10 ml·kg^−1^, and FiO_2_: 1.0 with isoflurane 1% to 1.5% (Liu et al., 2014; Pacher et al., 2008). Using an open chest approach via the RV apex, a Millar microtip catheter (PVR‐1045) connected to an MPVS Ultra Single Segment Pressure–Volume Unit and PowerLab (ADInstruments) was introduced to the RV cavity, and RV pressure and volume were simultaneously measured (Liu et al., 2014; Pacher et al., 2008; Tabima et al., 2010). After baseline recordings, measurements were repeated during brief occlusion of the inferior vena cava to alter the venous return. A catheter was calibrated for volume measurements in the blood and with the hypertonic saline dilution method (Liu et al., 2014; Pacher et al., 2008; Tabima et al., 2010). Parameters of RV function were derived from the baseline RV pressure–volume loops, including RV end‐systolic pressure, cardiac output, ejection fraction, stroke work, relaxation factor (*τ*), end‐diastolic elastance (*E*_ed_), and chamber compliance (*E*_ed_^−1^; Liu et al., 2014). Pressure–volume area (PVA; an estimate of myocardial O_2_ consumption), stroke work, and ventricular mechanical efficiency ([stroke work·PVA^−1^] × 100) were estimated based on the baseline RV pressure–volume relations (Liu et al., 2014). Load‐independent indices of systolic function such as preload‐recruitable stroke work and end‐systolic elastance (*E*_es_) were derived from pressure–volume loops with vena cava occlusion. To assess RV afterload and RV–PA coupling efficiency, effective arterial elastance (*E*_a_), and *E*_es_/*E*_a_ were calculated. Total pulmonary vascular resistance (tPVR = RV end‐systolic pressure/cardiac output) was used to estimate the resistance of pulmonary vasculature (Liu et al., 2014). Analysis was performed on LabChart software (v8.1.16, ADInstruments) by an investigator blinded to treatment assignments.

### 2.4 Assessment of RV hypertrophy

After excision of the heart, the RV wall was separated from the LV wall and the interventricular septum. The ratio of the RV weight to the weight of the left ventricle plus septum (i.e., Fulton index) was calculated as an index of RV hypertrophy (Beloiartsev et al., 2015).

### 2.5 RV collagen content assay

RV was fixed in 2% paraformaldehyde (w/v) and stained with Picrosirius red to determine interstitial collagen fractions as previously described (Lang et al., 2012). Image acquisition and analysis were performed by an investigator blinded to treatment assignments. Collagen content (red color) was digitally measured by systematically traversing the tissue slice using ImagePro software (Media Cybernetics) and presented for each tissue sample as the average of the percentage of fibrosis over the tissue area per field of view.

### 2.6 Immunohistochemistry

Immunohistochemistry was performed based on previously published methods with minor adjustments (Menon & Fisher, 2015). Paraffin‐embedded lung tissue sectioned at 4 μm thickness was deparaffinized in xylene and rehydrated in a series of solutions with different concentrations of ethanol, followed by washing with phosphate‐buffered saline (PBS). Antigen retrieval was performed by heating sections at 90°C in citrate buffer (10 mmol/L, pH 6.0) for 15 min, followed by washing with PBS‐Tween for 10 min, three times. The sections were blocked by BLOXALL® Endogenous Peroxidase and Alkaline Phosphatase Blocking Solution (Vector laboratories) for immunohistochemistry staining or 3% hydrogen peroxide for 30 min for immunofluorescent staining to eliminate the endogenous peroxidase activity, followed by nonspecific blocking with bovine serum albumin in PBS‐Tween (5%) for 60 min at room temperature.

To assess muscularization of the pulmonary microvasculature, sections of lung tissue were stained with an antibody against α‐smooth muscle actin (α‐SMA; 1:300 for 1 h, Ab124964, Abcam) using the ImmPRESS Horse Anti‐Rabbit IgG PLUS Polymer Kit Peroxidase (MP‐7801, Vector Laboratories). The sections were counterstained with hematoxylin and eosin solution.

For proliferating cell nuclear antigen (PCNA) staining in pulmonary microvasculature, lung tissue sections were incubated with rabbit anti‐PCNA antibody (1:300, ab252848, Abcam) and mouse anti‐α‐SMA conjugated with Alexa fluor 488 (1:250, F3777, Sigma‐Aldrich) at 4°C overnight. 4′,6‐diamidino‐2‐phenylindole (DAPI) was used to stain the nuclei. To eliminate background autofluorescence, Vector TrueVIEW® Autofluorescence Quencher Kit (SP‐8400‐15, Vector Lab) was used per the manufacturer's protocol.

To assess for expression of β3 ARs in the lungs of mice, staining was performed with rabbit anti‐β3 AR antibody (1:300 for 1 h, ab76249, Abcam) and rat anti‐CD31 antibody (1:250 for 1 h, 14‐0311‐82, Invitrogen) to identify colocalization of β3 ARs with the endothelial layer of vessels. Human lung sections were incubated with rabbit anti‐β3 AR antibody (1:300 for 1 h, ab76249, Abcam) and counterstained with hematoxylin and eosin to identify vessels. Nonspecific IgG (1: 250, 2 μg, for 1 h, 02‐6102, Sigma‐Aldrich) was used as a negative control for both mouse and human lung samples.

All images were acquired at 20× magnification with a Hitachi HV F202 camera (bright field) and Hamamatsu Orca flash 4.0 camera (fluorescent) on an auto Zeiss Axio Scan.Z1 Slide Scanner (Zeiss). The whole tissue surface was scanned, and 10 fields of view randomly selected by using the software from Zeiss were imaged by an investigator blinded to group assignments. For bright field imaging, the exposure time was 5 s. For fluorescence imaging, the exposure time was 0.25 s for Alexa fluor 488, 0.1 s for DAPI, and 0.5 s for other fluorescent images (Lang et al., 2012).

Image analysis was performed by an investigator blinded to group assignments. To assess muscularization, intraacinar blood vessels (≥15 and <50 μm in external diameter) containing α‐SMA‐positive cells in their walls were counted (Beloiartsev et al., 2015). Quantification of positive PCNA colocalized with α‐SMA was performed by using ImagePro software (Media Cybernetics). Data are presented as a percentage of positive staining for both markers per field of view.

### 2.7 Western blot

Protein from lung tissue was extracted by radioimmunoprecipitation assay (RIPA) buffer (Ab156034, Abcam). Denatured proteins were resolved following the NuPAGE Bis‐Tris Mini Gel Electrophoresis protocol (NP0336BOX, NuPAGE 4%–12% Bis‐Tris Protein Gels, Thermo Scientific), transferred onto polyvinylidene difluoride membranes, and probed with primary antibodies overnight at 4°C. The primary antibodies and concentrations used were selected based on the selectivity of the target antigens according to previous publications and manufacturers' instructions. Given the relative lack of selectivity of commercial antibodies for β3 ARs, several antibodies were examined using negative and positive controls, and antibodies that were most selective for mouse and human tissue were chosen.

The primary antibodies used included rabbit anti‐sGCα1 antibody (1:2000, ab50358, Abcam); rabbit anti‐sGCβ1 antibody (1:2000, ab154841, Abcam); rabbit anti‐eNOS antibody (1:2000, ab199956, Abcam); muscle actin (1:2000, ab136812, Abcam); goat anti‐β3 AR antibody (1:1000, M‐20, sc‐1473, Santa Cruz); rabbit anti‐β3 AR antibody (1:2000, ab76249, Abcam); and rabbit anti‐β‐actin antibody (1:5000, ab115777, Abcam). Following incubation with horseradish peroxidase‐conjugated secondary antibodies (1:5000, anti‐rabbit and antimouse, Abcam and Bio‐red), blots were developed with an enhanced chemiluminescence kit (34,075, Thermo Scientific). Imaging was performed using ChemiDoc Imaging System (Australian BioResources). Exposure times were adjusted to ensure that the variation in signal intensity was in the linear dynamic range and was kept consistent for each target protein. Analysis was performed using ImageJ (National Institutes of Health) software by an investigator blinded to treatment assignments. The expression of proteins was quantified by densitometry as the ratio of target protein divided by β‐actin used as a loading control.

### 2.8 Immunodetection of eNOS glutathionylation

Glutathionylation of eNOS was detected in tissue homogenates by coimmunoprecipitation using the previously published methods (Karimi Galougahi, Liu, et al., 2016). Briefly, the tissue homogenate (1 mg protein) was incubated with 2.5 μg anti‐eNOS antibody (AF950, R&D) or with 2.5 μg IgG control (02‐6202, Sigma‐Aldrich) for 1 h at 4°C and then with protein A/G‐Plus agarose beads (SCZ‐SC‐2003, Santa Cruz Biotechnology Inc.) for 1 h at 4°C. The beads were then collected by centrifugation at 1509 *g* for 30 s and washed in RIPA buffer four times. The proteins bound to the collected beads were then eluted in 40 μl of 2X Laemmli buffer by boiling the samples at 95°C for 5 min, followed by centrifugation at 1509 *g* for 30 s at 4°C. The collected protein was subjected to SDS‐PAGE and probed with an antiglutathione antibody (1: 1000, 101‐A‐250, ViroGen) overnight at 4°C. The same steps mentioned for other Western blots were followed for the development and analysis of the immunoblots by an investigator blinded to treatment assignments.

### 2.9 High‐performance liquid chromatography analysis of dihydroethidium oxidation products

Immediately after harvesting from mice, a piece of lung was incubated in PBS containing the metal chelator diethylenetriaminepentaacetic acid (100 μmol/L) to minimize artificial oxidation and dihydroethidium (DHE, 50 μmol/L) in 1.5 ml Eppendorf tubes (37°C, 30 min, in the dark) as previously described (Michel et al., 2020). Separation of the $O_{2}^{\cdot -}$‐dependent 2‐hydroxy‐ethidium (2‐OH‐E+) product from the nonspecific product ethidium (E+) was performed (Zielonka et al., 2008) by an investigator blinded to treatment assignments using Vanquish UHPLC coupled to TSQ Altis Triple Quadrupole Mass Spectrometer (Thermo Fisher Scientific). The quantified 2‐OH‐E+ levels were normalized to the DHE peak (to account for the DHE that has entered the tissue) and protein concentration (to account for the size of the tissue).

### 2.10 Statistics

Primary endpoint was a change in the RV systolic pressure by CL316243 in the Hx model. Based on the hemodynamic data for riociguat, and assuming a similar efficacy for CL316243, with a relative reduction of 32% in the RV systolic pressure from 46 mmHg (SD = 9; Dumitrascu et al., 2006), we calculated that *n* = 5 mice per group would provide 80% statistical power with *α* = 0.05 to detect a significant difference compared with the control hypoxic mice. We included an additional *n* = 1 per group to ensure that RV pressure–volume loop data with acceptable quality are acquired in an adequate number of mice based on previously published criteria (Liu et al., 2014; Pacher et al., 2008; Tabima et al., 2010, 2017). Data from all mice in which invasive hemodynamics were measured were included. Additionally, we included *n* = 2 mice per group to account for likely mortality due to multiple gavages over 14 days (Arantes‐Rodrigues et al., 2012), and *n* = 2 per group to ensure the adequacy of biospecimens for numerous molecular experiments that we planned to perform (i.e., total *n* = 10 per group). A total number of mice per group for each experiment that is specified in the tables and figures reflects the aforementioned points. Data are presented as mean ± SD. The normality of data was assessed using Kolmogorov–Smirnov test. *p* values are from univariate ANOVA and post‐hoc Tukey's test or Kruskal–Wallis test with Dunn's multiple comparisons where appropriate. Unpaired Student's *t* test was used for comparison between the two groups. Two‐tailed tests were used. Statistical analysis was conducted using GraphPad Prism (version 9.0.0). Statistical significance was inferred at *p* < 0.05.

## 3 RESULTS

The data for the Hx and SHx models are presented in the tables and figures. All *p* values for differences between groups are reported in the tables and figures.

### 3.1 Echocardiographic indices

In both Hx and SHx models, RV wall thickness increased in hypoxic mice treated with vehicle compared with the normoxic control mice treated with vehicle (Tables 1 and 2). Treatment with CL316243, sildenafil, or riociguat decreased RV wall thickness compared with the hypoxic mice treated with vehicle. PAT decreased in hypoxic mice treated with vehicle, while treatment with CL316243, sildenafil, or riociguat increased PAT in both models. There were no differences in the LV dimensions or ejection fraction between the normoxic and hypoxic groups in both models (Tables 1 and 2).

**TABLE 1:** Echocardiographic indices in the hypoxia model

Echocardiographic indices	Normoxia + vehicle (*n* = 10)	Hypoxia + vehicle (*n* = 7)	Hypoxia + CL (*n* = 7)	Hypoxia + riociguat (*n* = 7)	Hypoxia + sildenafil (*n* = 5)
LV dimensions and contractility					
LVEDD, mm	3.8 ± 0.2	4.0 ± 0.3	3.8 ± 0.2	3.5 ± 0.2	3.8 ± 0.2
LVESD, mm	2.5 ± 0.1	2.8 ± 0.3	2.6 ± 0.2	2.4 ± 0.2	2.7 ± 0.1
LV ejection fraction, %	65 ± 2	60 ± 8	59 ± 2	63 ± 6	59 ± 5
RV dimensions and Doppler indices					
RV wall, mm	0.26 ± 0.02	0.59 ± 0.06*	0.41 ± 0.01^†^	0.38 ± 0.03^†^	0.39 ± 0.01^†^
Pulmonary acceleration time, ms	23 ± 0.9	13.3 ± 0.9*	17.6 ± 0.5^†^	18.3 ± 0.6^†^	19.4 ± 0.4^†^
Ejection time, ms	63.3 ± 1.9	57.7 ± 1.8	59.7 ± 1.3	59.5 ± 1.5	59.2 ± 1.9
Pulmonary acceleration time/ejection time	0.37 ± 0.01	0.23 ± 0.01*	0.29 ± 0.01^†^	0.31 ± 0.01^†^	0.33 ± 0.01^†^
Pulmonary velocity time integral, mm	25.7 ± 0.9	17.9 ± 0.7*	20.3 ± 0.8^†^	20.8 ± 0.7^†^	21.4 ± 1.5^†^

*Note*: *N* = 5–10 mice/group.

Abbreviations: CL, CL316243; LV, left ventricle; LVEDD, left ventricular end‐diastolic dimension; LVESD, left ventricular end‐systolic dimension; RV, right ventricle.

* *p* < 0.05 versus. normoxic controls.

^†^ *p* < 0.05 versus hypoxic controls.

**TABLE 2:** Echocardiographic indices in the Sugen hypoxia model

Echocardiographic indices	Normoxia + vehicle (*n* = 10)	SHx + vehicle (*n* = 7)	SHx + CL (*n* = 7)	SHx + riociguat (*n* = 5)	SHx + sildenafil (*n* = 7)
LV dimensions and contractility					
LVEDD, mm	3.8 ± 0.3	4.0 ± 0.3	3.9 ± 0.2	4.0 ± 0.3	3.9 ± 0.1
LVESD, mm	2.5 ± 0.2	2.7 ± 0.2	2.6 ± 0.2	2.6 ± 0.1	2.7 ± 0.1
LV ejection fraction, %	64 ± 5	60 ± 4	63 ± 4	64 ± 3	61 ± 4
RV dimensions and Doppler indices					
RV wall	0.26 ± 0.01	0.70 ± 0.06*	0.48 ± 0.03^†^	0.46 ± 0.05^†^	0.46 ± 0.03^†^
Pulmonary acceleration time, ms	23 ± 2.6	11.8 ± 0.6*	16.7 ± 0.5^†^	17.9 ± 0.7^†^	18.8 ± 0.7^†^
Ejection time, ms	61.6 ± 1.6	55.9 ± 3.0*	63.1.7 ± 2.5^†^	57.0 ± 3.0	56.9 ± 3.3
Pulmonary acceleration time/ejection time	0.38 ± 0.04	0.21 ± 0.02*	0.27 ± 0.01^†^	0.31 ± 0.02^†^	0.33 ± 0.03^†^
Pulmonary velocity time integral, mm	25.0 ± 0.7	16.9 ± 0.8*	19.1 ± 0.9^†^	19.8 ± 0.8^†^	21.9 ± 1.8^†^

*Note*: Values are means ± SD. *N* = 5–10 mice/group. SHx + CL group.

Abbreviations: CL, CL316243; LV, left ventricle; LVEDD, left ventricular end‐diastolic dimension; LVESD, left ventricular end‐systolic dimension; RV, right ventricle.

* *p* < 0.05 versus normoxic control.

^†^ *p* < 0.05 versus hypoxic control.

### 3.2 Invasive right ventricular hemodynamics

Representative pressure–volume loops for normoxic mice treated with vehicle and hypoxic mice treated with vehicle or CL316243 are shown in Figure 1. RV systolic pressure was significantly increased in both models compared with the normoxic mice (Tables 3 and 4), confirming the establishment of PAH. Treatment with CL316243, sildenafil, or riociguat reduced RV systolic pressure by a similar magnitude compared with the mice treated with vehicle in both models. RV stroke work and PVA increased in the hypoxic control mice compared with the normoxic controls. All treatments reduced both PVA and stroke work such that their ratio was not altered by therapies compared with the hypoxic control mice.

**TABLE 3:** Functional and hemodynamic parameters from the right ventricular pressure–volume loop analysis in the hypoxia model

Hemodynamic index	Normoxia + vehicle (*n* = 8)	Hypoxia + vehicle (*n* = 5)	Hypoxia + CL (*n* = 5)	Hypoxia + riociguat (*n* = 5)	Hypoxia + sildenafil (*n* = 5)
Heart rate, bpm	494 ± 35	485 ± 59	487 ± 29	484 ± 60	476 ± 42
RVSP, mmHg	21.3 ± 1.5	64.1 ± 6.7*	39.8 ± 1.3^†^	44.3 ± 5.5^†^	34.8 ± 1.0^†^
RVDP, mmHg	2.3 ± 0.4	6.6 ± 1.5*	4.6 ± 0.6^†^	4.0 ± 0.6^†^	4.5 ± 0.5^†^
Cardiac output, μl.min^−1^	8275 ± 368	4027 ± 609*	5219 ± 263^†^	4915 ± 108^†^	5123 ± 171^†^
Ejection fraction, %	61.0 ± 2.1	41.8 ± 2.8*	50.0 ± 2.0^†^	53.7 ± 2.1^†^	53.3 ± 2.5^†^
RV energetics					
PVA, mmHg·μl	512 ± 32	1084 ± 116*	718 ± 101^†^	677 ± 96^†^	698 ± 73^†^
Stroke work, mmHg·μl	312 ± 41	538 ± 22*	370 ± 45^†^	372 ± 43^†^	353 ± 36^†^
RV mechanical efficiency, %	61 ± 5	50 ± 3*	51 ± 2	55 ± 3	50 ± 2
RV afterload					
*E*_a_, mmHg·μl^−1^	1.3 ± 0.1	7.8 ± 1.1*	3.7 ± 0.2^†^	4.3 ± 0.1^†^	3.2 ± 0.3^†^
tPVR, mmHg·min·ml^−1^	2.6 ± 0.2	16.3 ± 3.7*	7.6 ± 0.4^†^	9.5 ± 1.3^†^	6.8 ± 0.3^†^
Load‐independent RV contractility					
*E*_es_, mmHg·μl^−1^	1.3 ± 0.3	4.6 ± 0.4*	2.9 ± 0.4^†^	3.0 ± 0.3^†^	2.7 ± 0.5^†^
PRSW, mmHg	9.2 ± 1.7	23.2 ± 3.8*	14.4 ± 0.6^†^	14.4 ± 2.7^†^	11.1 ± 0.8^†^
RV diastolic function					
*E*_ed_, mmHg·μl^−1^	0.9 ± 0.3	3.5 ± 0.6*	1.2 ± 0.2^†^	2.4 ± 0.7^†^	1.8 ± 0.6^†^
Tau, ms	8.1 ± 2.9	11.1 ± 2.1	8.3 ± 1.0	11.4 ± 1.1	13.2 ± 3.0
Chamber compliance, μl·mmHg^−1^	1.3 ± 0.6	0.3 ± 0.0*	0.7 ± 0.1	0.4 ± 0.1	0.6 ± 0.2

*Note*: *N* = 5–8 mice/group.

Abbreviations: CL, CL316243; *E*_a_, arterial elastance; *E*_es_, end‐systolic elastance; PRSW, preload‐recruitable stroke work; PVA, pressure–volume area; RVDP, right ventricular diastolic pressure; RVSP, right ventricular systolic pressure; tPVR, total pulmonary vascular resistance.

* *p* < 0.05 versus normoxic controls.

^†^ *p* < 0.05 versus hypoxic controls.

**TABLE 4:** Invasive hemodynamic measurements in the Sugen hypoxia model

Hemodynamic index	Normoxia + vehicle (*n* = 7)	SHx + vehicle (*n* = 5)	SHx + CL (*n* = 7)	SHx + Riociguat (*n* = 5)	SHx + sildenafil (*n* = 7)
Heart rate, bpm	492 ± 38	469 ± 25	466 ± 25	469 ± 28	497 ± 15
RVSP, mmHg	21.3 ± 1.5	83.8 ± 6.3*	60.6 ± 4.0^†^	58.4 ± 10.9^†^	66.5 ± 2.1^†^
RVDP, mmHg	2.6 ± 0.8	6.3 ± 1.3*	6.2 ± 1.9	4.3 ± 1.3	6.1 ± 0.5
Cardiac output, μl.min^−1^	8275 ± 368	4004 ± 83*	4156 ± 326	4217 ± 358	4647 ± 126
Ejection fraction, %	61 ± 2	26 ± 6*	38 ± 5^†^	38 ± 5^†^	39 ± 3^†^
RV afterload					
*E*_a_, mmHg·μl^−1^	1.3 ± 0.1	9.9 ± 1.4*	6.9 ± 0.9^†^	6.5 ± 1.2^†^	7.1 ± 0.3^†^
tPVR, mmHg·min·ml^−1^	2.6 ± 0.2	21.3 ± 3.8*	14.7 ± 1.8^†^	13.9 ± 2.4^†^	14.6 ± 0.8^†^
Load‐independent RV contractility					
*E*_es_, mmHg·μl^−1^	1.3 ± 0.3	5.2 ± 1.2*	4.7 ± 0.6	4.6 ± 0.9	4.9 ± 0.7
PRSW, mmHg	9.2 ± 1.7	43 ± 7*	28.0 ± 7.8^†^	24.0 ± 4.5^†^	33.8 ± 3.4
RV–PA coupling efficiency					
*E*_es_/*E*_a_	0.98 ± 0.13	0.52 ± 0.05*	0.69 ± 0.06^†^	0.71 ± 0.07^†^	0.69 ± 0.08^†^
RV diastolic function					
*E*_ed_, mmHg·μl^−1^	0.9 ± 0.2	4.8 ± 0.6*	1.5 ± 0.2^†^	1.5 ± 0.2^†^	2.3 ± 0.3^†^
Tau, ms	8.2 ± 3.0	7.7 ± 1.0	7.7 ± 0.8	6.8 ± 1.0	7.6 ± 3.0
Chamber compliance, μl·mmHg^−1^	1.3 ± 0.6	0.2 ± 0.0*	0.7 ± 0.1	0.7 ± 0.1	0.4 ± 0.1

*Note*: Values are means ± SD. *N* = 5–7 mice/group.

Abbreviations: CL, CL316243; *E*_a_, arterial elastance; *E*_es_, end‐systolic elastance; PRSW, preload recruitable stroke work; PVA, pressure–volume area; RVDP, right ventricular diastolic pressure; RVSP, right ventricular systolic pressure; tPVR, total pulmonary vascular resistance.

* *p* < 0.05 versus normoxic control.

^†^ *p* < 0.05 versus hypoxic control.

In both models, RV afterload (*E*_a_ and tPVR) increased in the hypoxic mice treated with vehicle compared with the normoxic control mice (Tables 3 and 4), and treatment with CL316243, ricocigaut, or sildenafil reduced the RV afterload compared with the hypoxic control mice.

**FIGURE 2:**
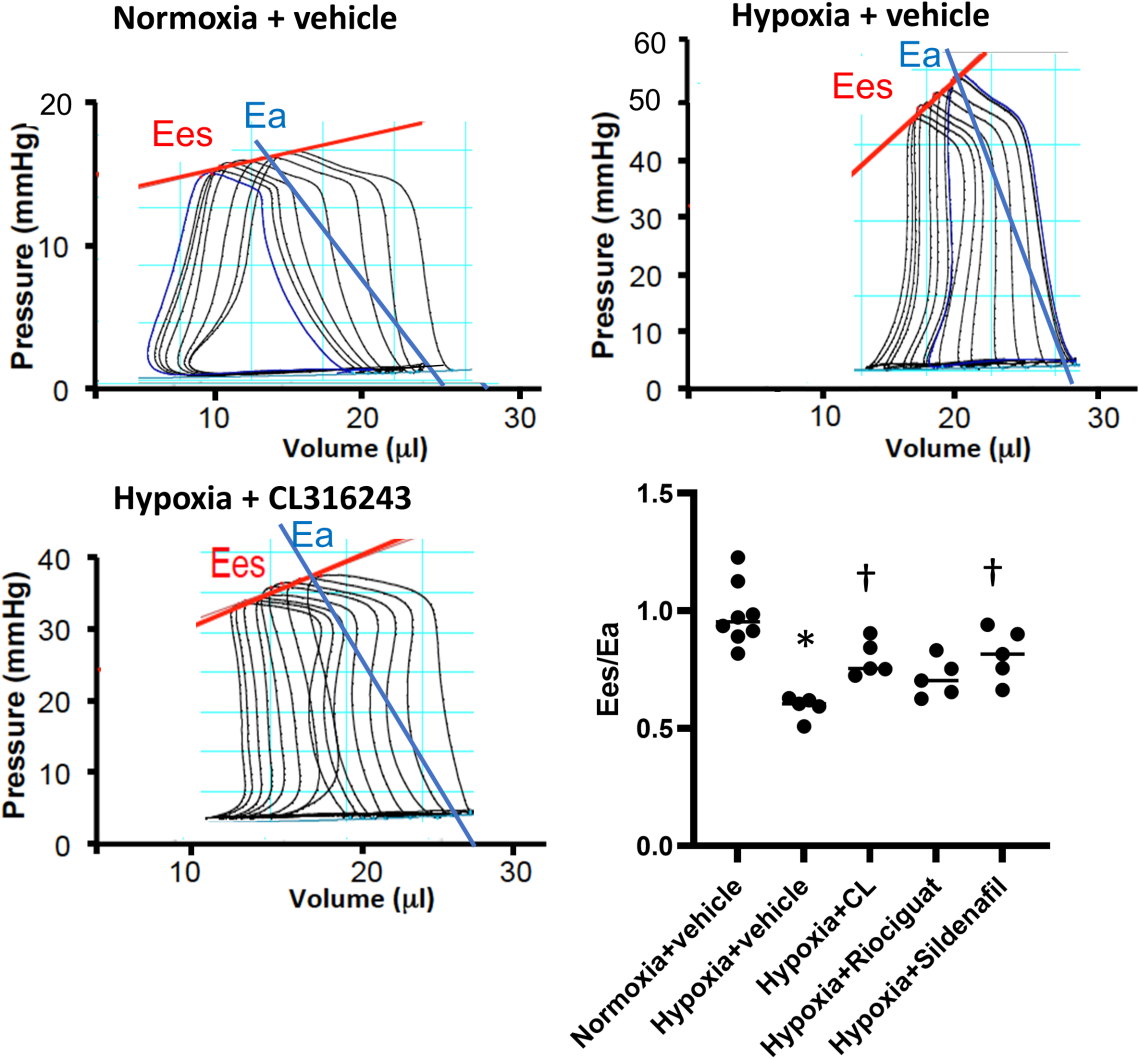
Pressure–volume loops and right ventricular (RV)–pulmonary artery coupling efficiency in the hypoxia model. Representative RV pressure–volume loops in a normoxic mouse treated with vehicle, a hypoxic mouse treated with vehicle, and a hypoxic mouse treated with CL316243 are shown. Calculation of end‐systolic elastance (*E*_es_) and effective arterial elastance (*E*_a_) is performed by decreasing preload via temporary occlusion of the inferior vena cava. *E*_es_ is determined from a linear approximation to the end‐systolic pressure–volume relationship, whereas *E*_a_ is the slope of the line that connects the ventricular end‐systolic point to the ventricular end‐diastolic volume projected on the volume axis. In hypoxic mice, *E*_es_ increased in response to increased *E*_a_, but as shown in the panel, this adaptation was inadequate, and *E*_es_/*E*_a_ ratio significantly dropped compared with the normoxic mice. Treatment with CL3106243 or sildenafil resulted in a significant increase in the *E*_es_/*E*_a_ ratio compared with the hypoxic control mice. *N* = 5–8 mice per group; *p < 0.0001 compared with normoxic controls, ^†^ *p* < 0.05 compared with hypoxic controls on univariate ANOVA.

To compensate for the increase in the RV afterload, RV contractility, as measured by the preload‐independent contractility indices (*E*_es_ and PRSW), increased in hypoxic mice in both models (Tables 3 and 4). The increase in the contractility was not adequate to effectively maintain RV–PA coupling efficiency in hypoxic mice treated with vehicle in both models, with the *E*_es_/*E*_a_ ratio significantly dropping compared with the noromoxic controls (Figure 2, Table 4). Treatment with CL316243 or sildenafil in the Hx model, and all treatments in the SUHx model, significantly increased *E*_es_/*E*_a_ ratio compared with control hypoxic mice, suggesting improved RV–PA coupling efficiency by treatments (Figure 2, Table 4). Consistent with the reduced RV afterload and enhanced RV–PA coupling efficiency, treatments increased RV ejection fraction (Tables 3 and 4).

All treatments resulted in reductions in the RV diastolic pressure in the Hx model (Table 3), whereas in the SUHx model, RV diastolic pressure was not significantly impacted by therapies (Table 4). Of the indicators of RV diastolic function, Eed was significantly increased while chamber compliance but not τ was significantly decreased in the control mice in both models (Tables 3 and 4). All treatments significantly reduced Eed compared with the hypoxic controls treated with vehicle in both models, but τ and chamber compliance were unchanged.

### 3.3 Right ventricular and pulmonary vascular remodeling

**FIGURE 3:**
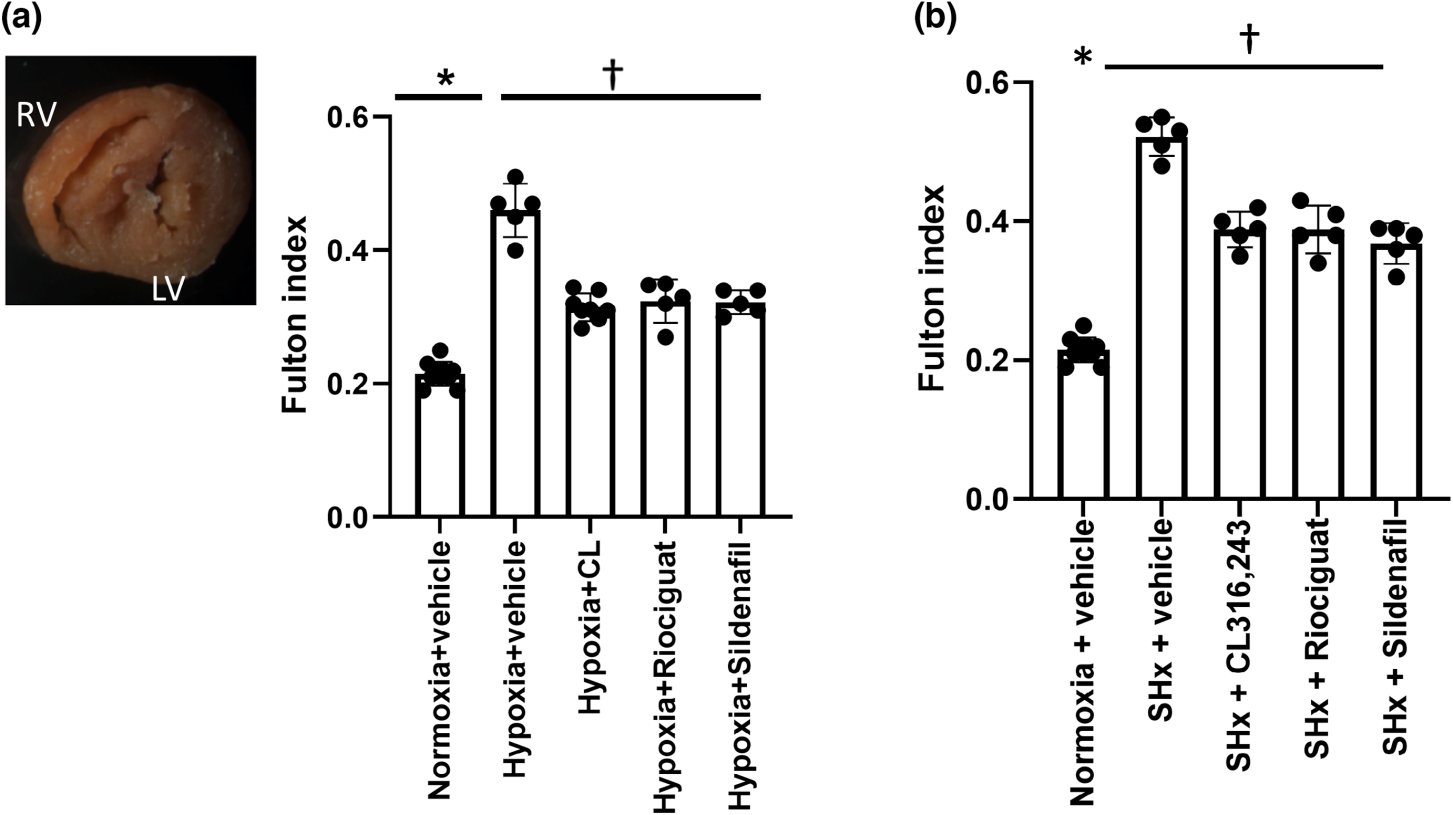
Effects of treatments on right ventricular hypertrophy. (a) In the hypoxia model. Hypoxia increased RV hypertrophy as assessed by Fulton's index. All treatments led to a significant reduction in RV hypertrophy, reflecting the improved RV–PA coupling efficiency and homeometric reverse remodeling of RV. *N* = 5–10 mice per group; *p < 0.0001 compared with normoxic controls, ^†^ *p* < 0.0001 compared with hypoxic controls on univariate ANOVA. (b) In Sugen hypoxia model. Right ventricular hypertrophy was significantly reduced by all treatments compared with the Sugen hypoxia control mice treated with vehicle. *N* = 5–8 mice/group; *p* < 0.05 versus normoxic control; ^†^ *p* < 0.05 versus hypoxic control on univariate ANOVA.

**FIGURE 4:**
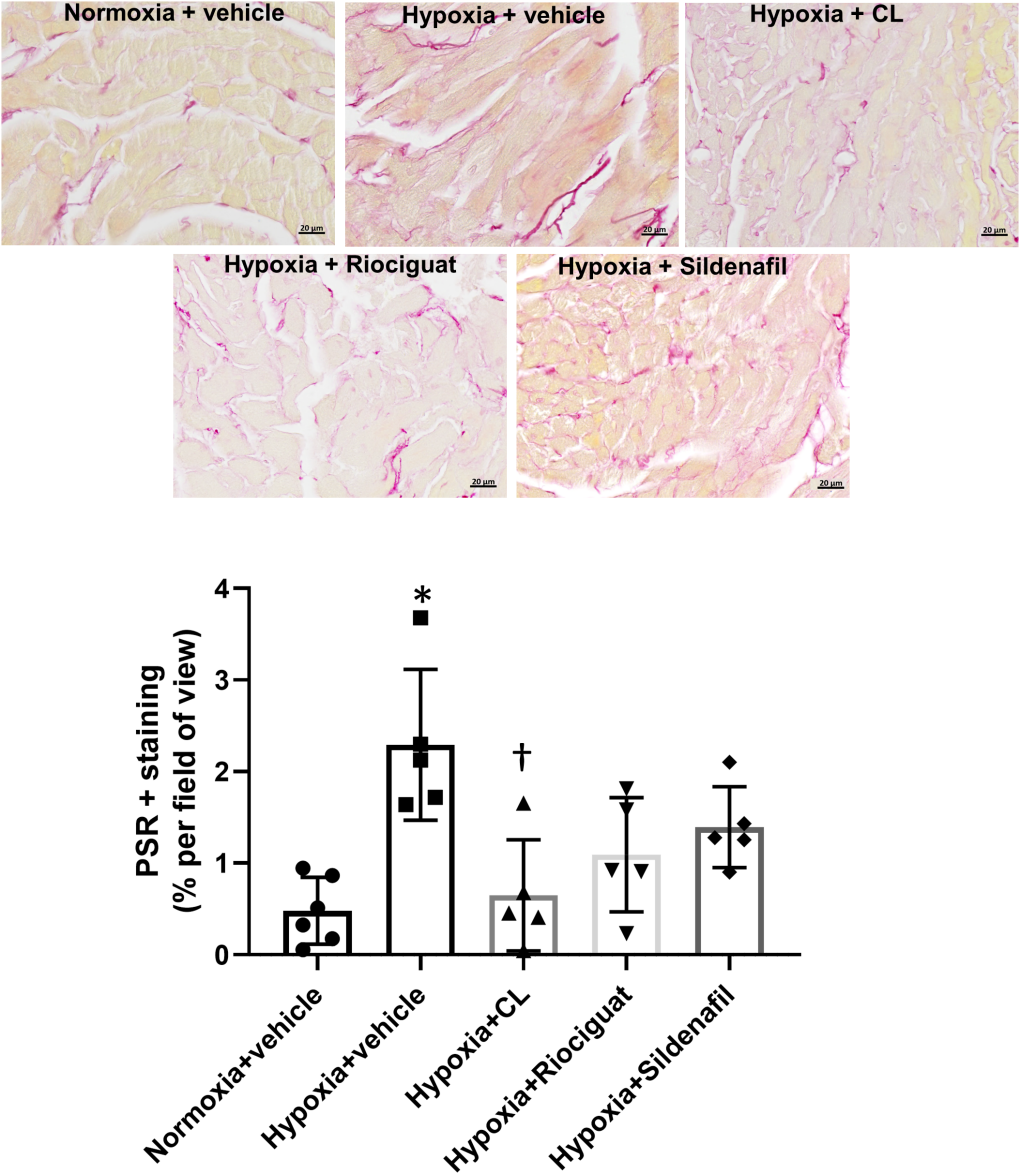
Collagen content in the right ventricle in the hypoxia model. Representative right ventricular sections stained with Picrosirius red (PSR) are shown. Collagen content (red color, expressed as a percentage of the area per field of view) was increased in the hypoxic mice treated with vehicle compared with normoxic mice treated with vehicle. Treatment with CL316243 (CL) reduced the collagen content compared with the hypoxic control mice. *N* = 5–6 mice/group, *p = 0.0008 compared with normoxic controls, ^†^ *p* < 0.05 compared with hypoxic controls on univariate ANOVA.

The Fulton index increased in hypoxic mice treated with vehicle in both models, indicating RV hypertrophy (Figure 3). There was no difference in the LV + septum weight between the groups (data not shown), confirming the differences in the Fulton index were due to changes in the RV weight. All treatments in both models resulted in attenuation of the RV hypertrophy, reflecting the improved matching of ventricular–vascular elastance. Collagen content in the RV was higher in the Hx mice treated with vehicle compared with the normoxic mice treated with vehicle (Figure 4). Treatment with CL316243, and not riociguat or sildenafil, reduced the collagen content in the RV compared with the control hypoxic mice (Figure 4).

**FIGURE 5:**
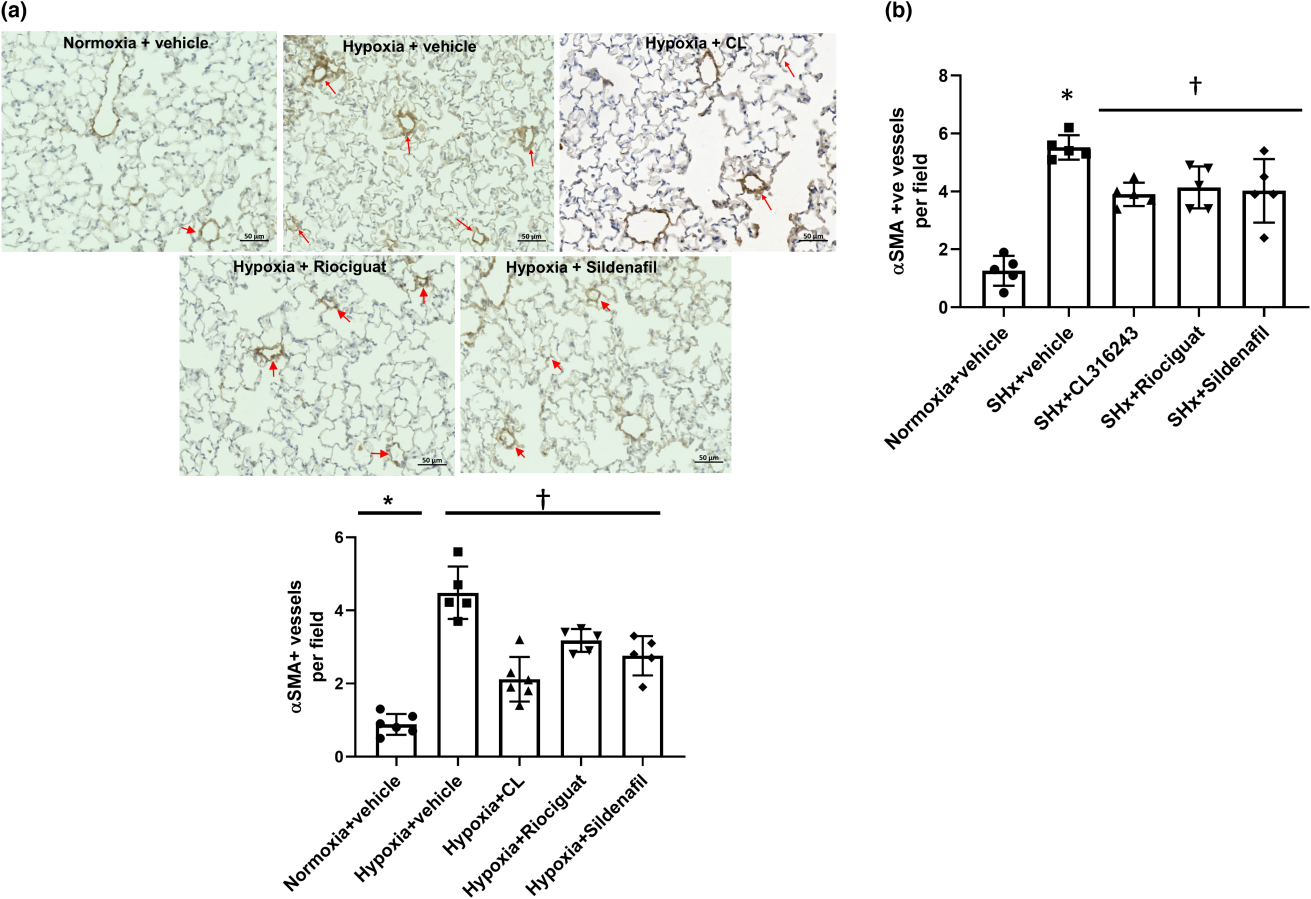
Effects of treatments on pulmonary vascular remodeling. (a) In the hypoxia model. Representative images of pulmonary vasculature are shown. Arrows point to α‐smooth muscle actin (SMA)‐positive alveolar vessels. The number of α‐SMA‐positive intraacinar vessels was higher in hypoxic mice treated with vehicle compared with the normoxic control mice. Treatments resulted in a significant reduction in the number of α‐SMA‐positive vessels compared with the hypoxic control mice. *N* = 5–6 mice/group. *p < 0.0001 compared with normoxic controls, ^†^ *p* < 0.05 compared with hypoxic controls on univariate ANOVA. (b) In Sugen hypoxia model. The number of α‐SMA‐positive vessels in the pulmonary microvasculature was significantly increased in the Sugen hypoxia control mice and was significantly reduced by treatments compared with vehicle. SHx, Sugen hypoxia, α‐SMA = α‐smooth muscle actin. *N* = 5 mice/group; *p* < 0.05 vs. normoxic control; ^†^ *p* < 0.05 versus hypoxic control on univariate ANOVA.

In both models, there was an increase in the number of muscularized arteries in the pulmonary microvasculature in the hypoxic control mice treated with vehicle compared with the normoxic control mice (Figure 5). All treatments significantly decreased the number of muscularized arteries compared with vehicle in both models.

### 3.4 Pulmonary vascular cell proliferation

**FIGURE 6:**
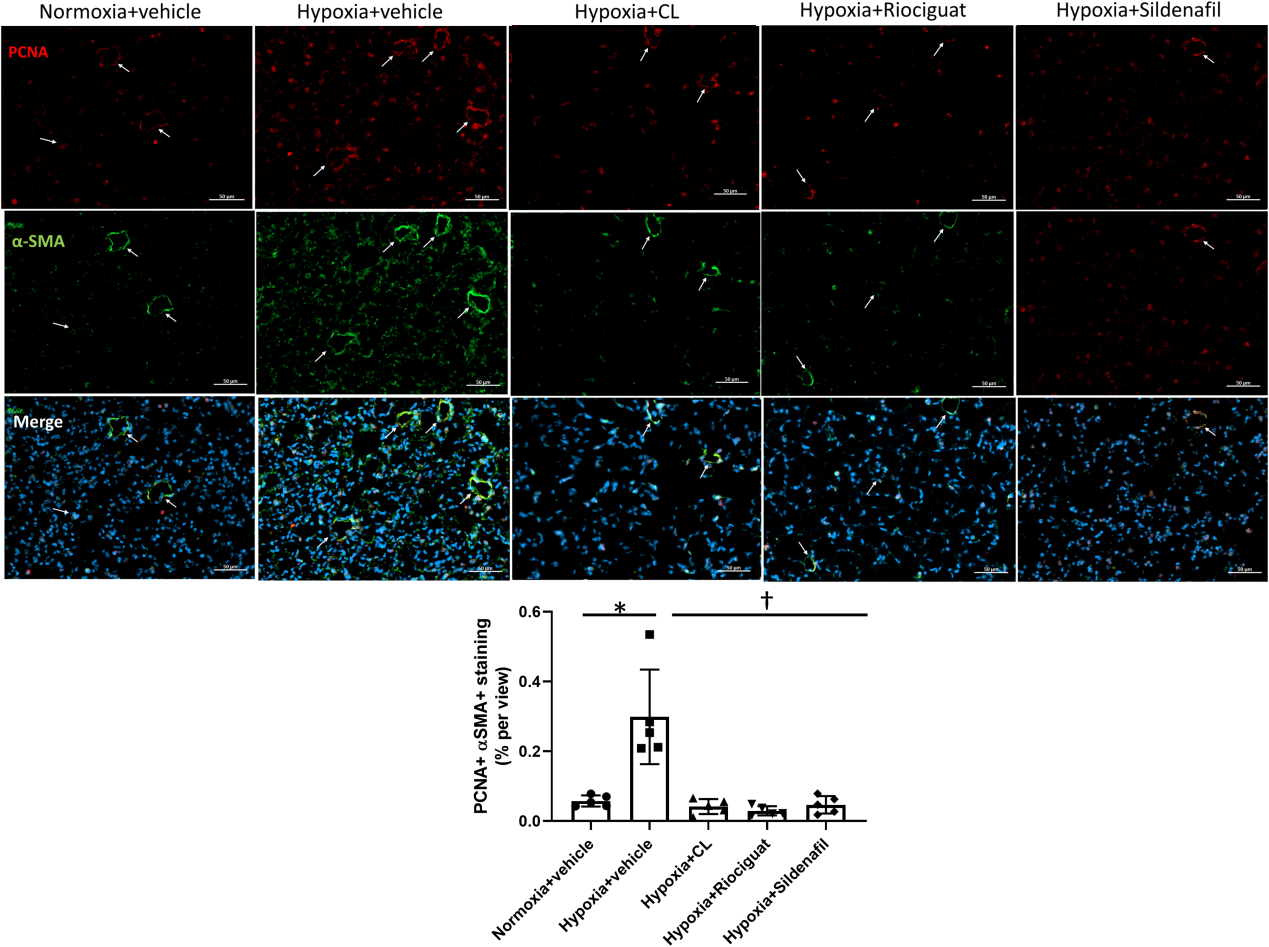
Effects of treatments on cell proliferation in the lungs of hypoxic mice. Representative figures for proliferating cell nuclear antigen (PCNA; red), α‐smooth muscle actin (SMA; green) immunostaining, and a summary of the data for different treatment groups are shown. Hypoxia increased the proliferation index in the α‐SMA‐positive cells in the lungs of mice treated with vehicle, while the proliferation index was reduced by all treatments. *N* = 5 mice/group, *p = 0.0007 compared with normoxic controls, ^†^ *p* < 0.001 compared with the hypoxic controls on univariate ANOVA.

Immunoreactivity for PCNA in cells that were positive for α‐SMA was significantly increased in the lungs of hypoxic mice compared with the healthy controls (Figure 6). The index of proliferation was significantly reduced in hypoxic mice that received CL316243, sildenafil, or riociguat compared with hypoxic mice treated with vehicle.

### 3.5 Expression of β3 ARs in the endothelium of pulmonary arteries

**FIGURE 7:**
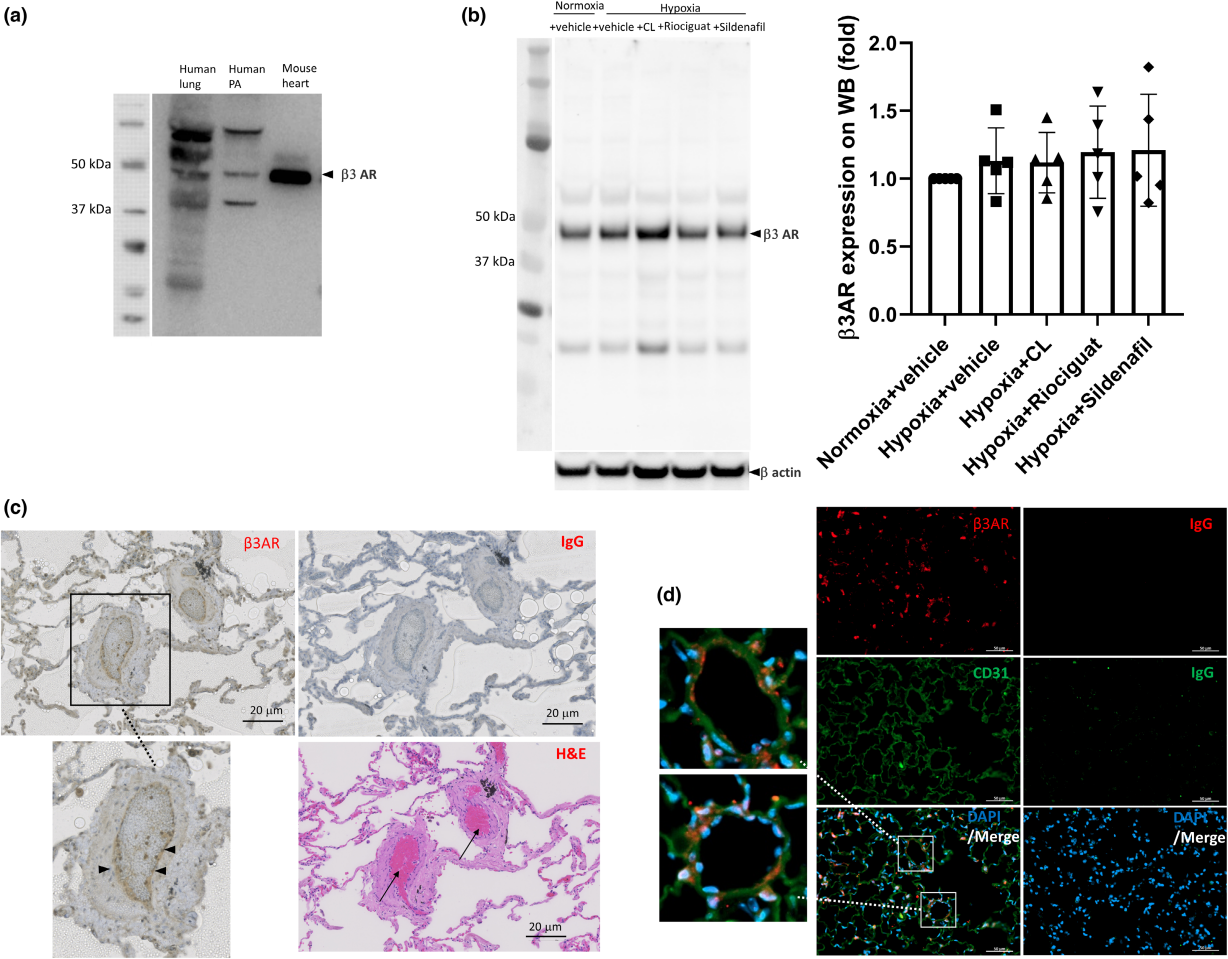
Expression of β3 adrenergic receptor. (a) In the lungs and pulmonary arteries of humans. Immunoblots of β3 adrenergic receptor (β3 AR) in the lung and pulmonary artery of humans. A mouse heart, with a higher amount of protein loaded compared with the human samples in the gel, was used as a positive control, with the band at this molecular weight previously shown by us to be selective for β3 adrenergic receptor (Bundgaard et al., 2010). (b) In the lungs of hypoxic mice. Immunoblots of the β3 AR in the lungs of mice are shown. β‐Actin was used as the loading control. Mean densitometries for the bands detected at ~44 kDa normalized to β‐actin are shown in the panel. Expression of β3 AR did not change in the lungs of hypoxic mice, with or without treatment, compared with the normoxic control mice. *N* = 5 mice/group. *p* = 0.67 compared with the normoxic controls on the Kruskal–Wallis test. WB = Western blot. (c) In the pulmonary vessels of humans. Immunostaining with β3 AR antibody in the human lung sections demonstrates expression of the receptor in the endothelial layer of vessels (arrowheads in inset, which is a vessel selected for higher magnification). The signal was absent in the sections immunostained with a nonspecific IgG antibody that was used as a negative control. Hematoxylin and eosin (H&E) staining was used to identify vessels containing the three layers of intima, media, and adventitia and filled with red blood cells (arrows). The scale bar represents 20 μm. (d) In the pulmonary vessels of mice. Co‐immunostaining with the endothelial marker cluster of differentiation 31 (CD31; red), β3 AR (green), and nuclei with DAPI (blue) demonstrates expression of the receptor in the endothelial layer of the pulmonary microvasculature (insets are two vessels selected for higher magnification). Staining with a nonspecific IgG was used as a negative control. The scale bar represents 50 μm.

Immunoblots for β3 AR showed expression of the receptor in the lung and pulmonary artery of humans (Figure 7a). Expression of β3 ARs did not change in the lungs of hypoxic mice compared with the normoxic controls (Figure 7b). Treatments did not change the expression of the receptor in the lungs of hypoxic mice (Figure 7b). Immunohistochemistry of human pulmonary arteries (Figure 7c) and co‐staining with the endothelial marker CD31 and β3 AR of mouse lung (Figure 7d) demonstrated expression of the receptor in the endothelium of pulmonary microvasculature.

### 3.6 eNOS expression, eNOS glutathionylation, and soluble guanylyl cyclase expression

**FIGURE 8:**
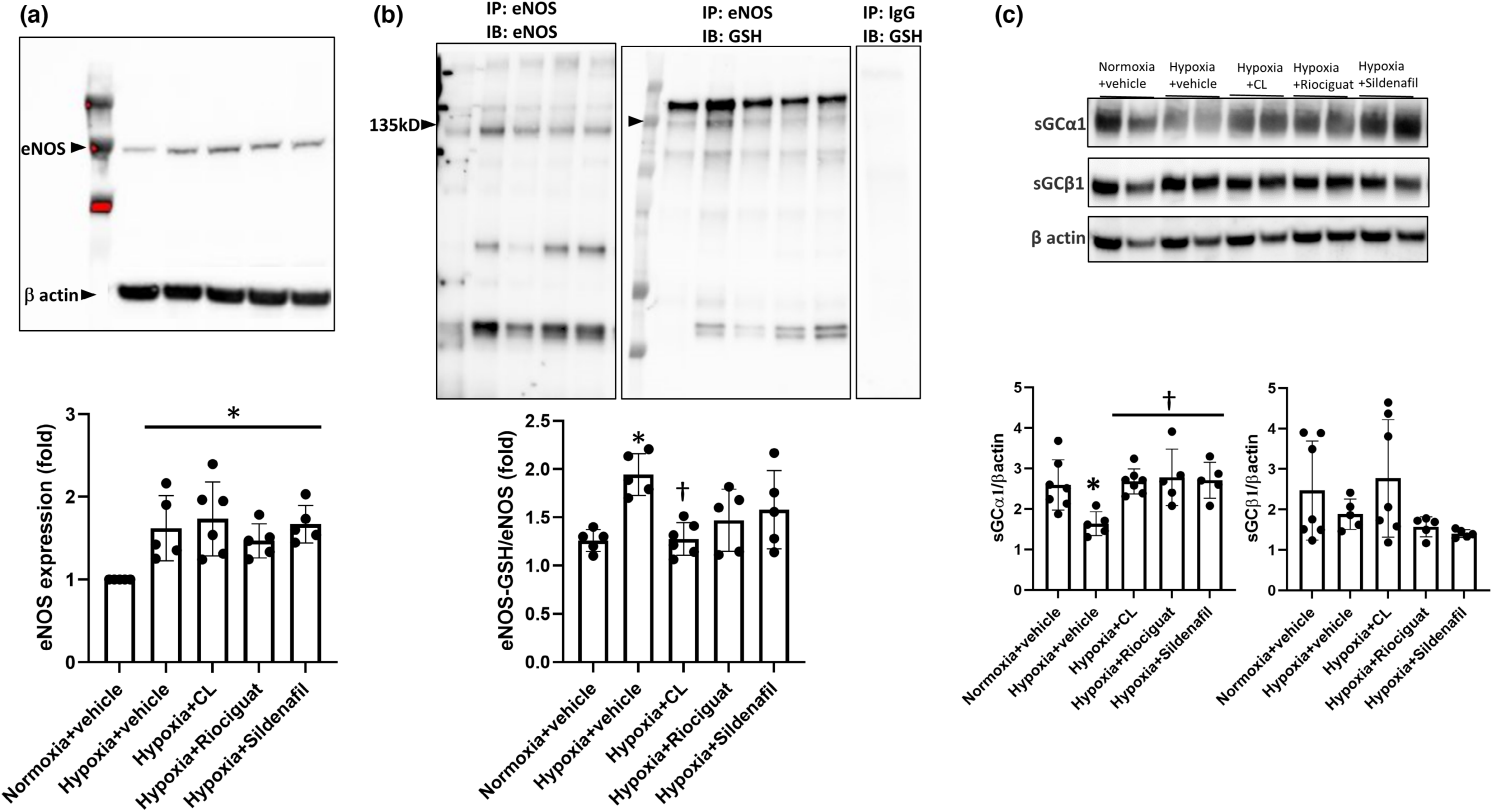
Effects of treatments on expression and glutathionylation of endothelial nitric oxide synthase and soluble guanylyl cyclase subunits in the lungs of hypoxic mice. (a) Expression of endothelial nitric oxide synthase. Immunoblots of endothelial nitric oxide synthase (eNOS) in the lungs are shown. β‐Actin was used as the loading control. Mean densitometries for eNOS normalized to β‐actin are shown in the panel. Expression of eNOS was higher in the lungs of hypoxic mice compared with the normoxic control mice. *N* = 5–6 mice. *p = 0.008 compared with the normoxic controls on Kruskal–Wallis test. (b) Glutathionylation of endothelial nitric oxide synthase. Immunoblots (IB) of eNOS and GSH performed on eNOS immunoprecipitate (IP) from the lungs of mice are shown. IP of a nonspecific IgG with IB for GSH was used as a negative control. Mean densitometries for GSH IB normalized to eNOS IB are shown in the panel. Glutathionylation of eNOS (eNOS‐GSH) was increased in the lungs of hypoxic mice treated with vehicle compared with the normoxic controls. Treatment with CL316243 reduced eNOS‐GSH compared with the hypoxic control mice. *N* = 5–6 mice. *p = 0.009 compared with the normoxic controls and ^†^ *p* < 0.02 compared with the hypoxic controls on Kruskal–Wallis test. (c) Expression of soluble guanylyl cyclase (sGC) subunits. Immunoblots of sGC α1 and β1 subunits are shown. β‐Actin was used as the loading control. Mean densitometries for sGC α1 and β1 subunits normalized to β‐actin are shown in the panels. Expression of sGC α1, not β1 subunit, was reduced in the lungs of hypoxic mice compared with the normoxic controls. All treatments increased the expression of sGC α1 compared with the hypoxic controls. *N* = 5–7 mice. *p = 0.03 compared with the normoxic controls, ^†^ *p* < 0.03 compared with the hypoxic controls on univariate ANOVA.

Expression levels of eNOS were higher in the lungs of hypoxic mice treated with vehicle or active treatments compared with the normoxic controls (Figure 8a). Treatment with CL316243, riociguat, or sildenafil did not change the eNOS expression levels compared with the hypoxic control mice. Glutathionylation of eNOS, an oxidative modification that results in reversible uncoupling of eNOS (Chen et al., 2010), was significantly increased in the lungs of hypoxic mice treated with vehicle compared with the normoxic control mice, as assessed by co‐immunopreciptation of eNOS and glutathionylated proteins normalized to eNOS expression (Figure 8b).

Treatment with CL316243 decreased eNOS glutathionylation compared with the hypoxic controls, suggesting that the treatment led to a reversal in the uncoupling of eNOS. Treatment with sildenafil or riociguat did not significantly change the eNOS glutathionylation levels compared with the hypoxic control mice. Moreover, expression of sGCα1 decreased in the hypoxic control mice while expression of sGCβ1 was not affected (Figure 8c). All treatments led to increased expression of sGCα1, but not sGCβ1, in the lungs compared with the hypoxic mice treated with vehicle.

### 3.7 Oxidative stress

**FIGURE 9:**
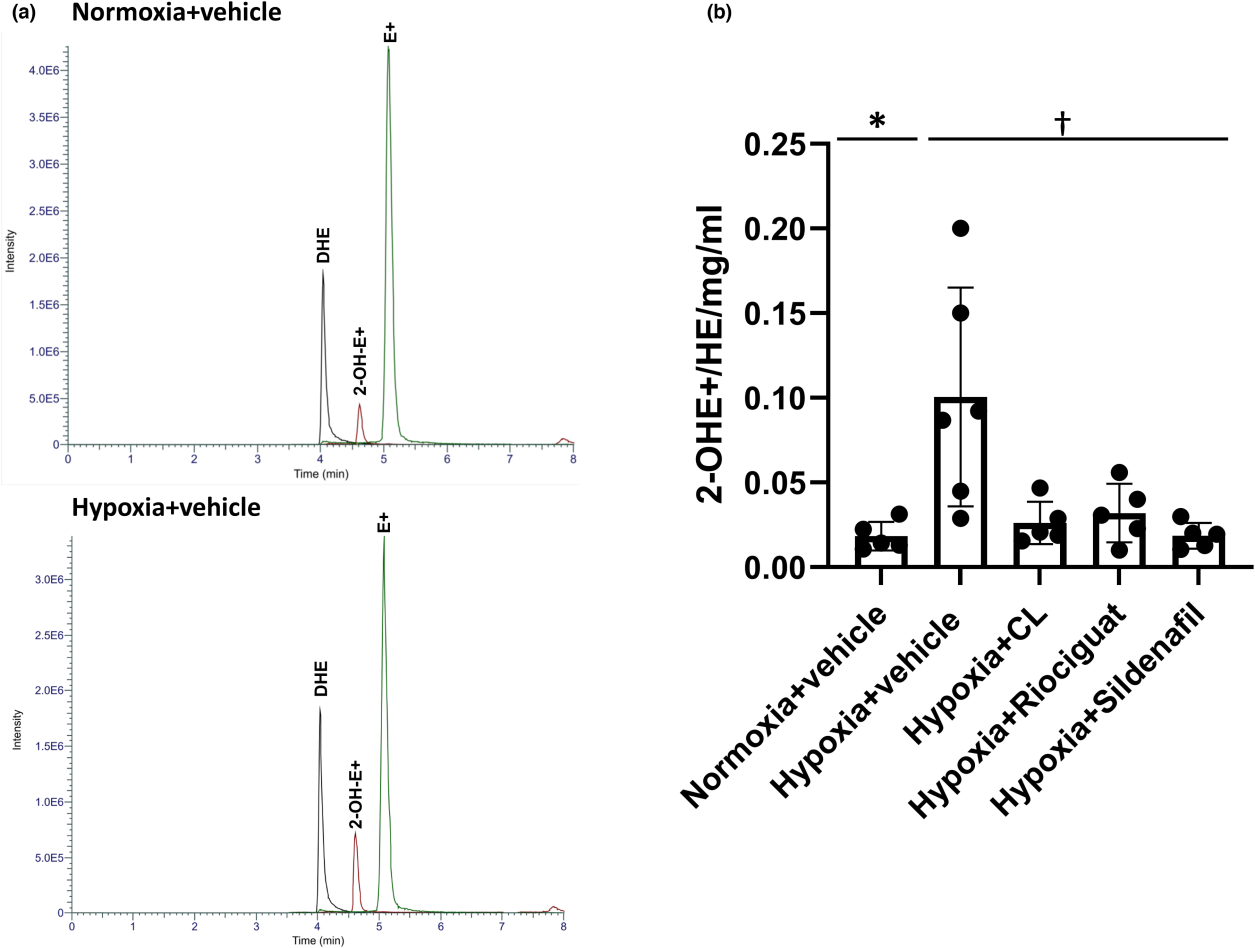
$O_{2}^{\cdot -}$ levels in the lungs by HPLC analysis of dihydroethidium oxidation products. (a) Representative chromatograms are chromatograms from a normoxic control and a hypoxic control mouse. On HPLC, the peaks for the specific (2‐OH‐E^+^) and nonspecific products (E^+^) resulting from the oxidation of dihydroethidium (DHE) are detected. (b) $O_{2}^{\cdot -}$ levels in the lungs. Mean values of 2‐OH‐E^+^ normalized over protein concentration are shown in the panel. Hypoxia increased $O_{2}^{\cdot -}$ levels in the lungs compared with the normoxic controls. Treatments led to a significant reduction in $O_{2}^{\cdot -}$ levels compared with the hypoxic control mice. *N* = 5–6 control, *p = 0.002 compared with the normoxic control, ^†^ *p* < 0.03 compared with the hypoxic control on univariate ANOVA. CL, CL316243.

Representative chromatograms from high‐performance liquid chromatography of the DHE oxidation products are shown in Figure 9a. There was a significant increase in $O_{2}^{\cdot -}$ levels in the lungs of hypoxic mice as measured by 2‐OH‐E+ levels, the specific product of DHE oxidation by $O_{2}^{\cdot -}$ (Figure 9b). All treatments resulted in a reduction in the 2‐OH‐E+ levels compared with the hypoxic control mice.

## 4 DISCUSSION

**FIGURE 10:**
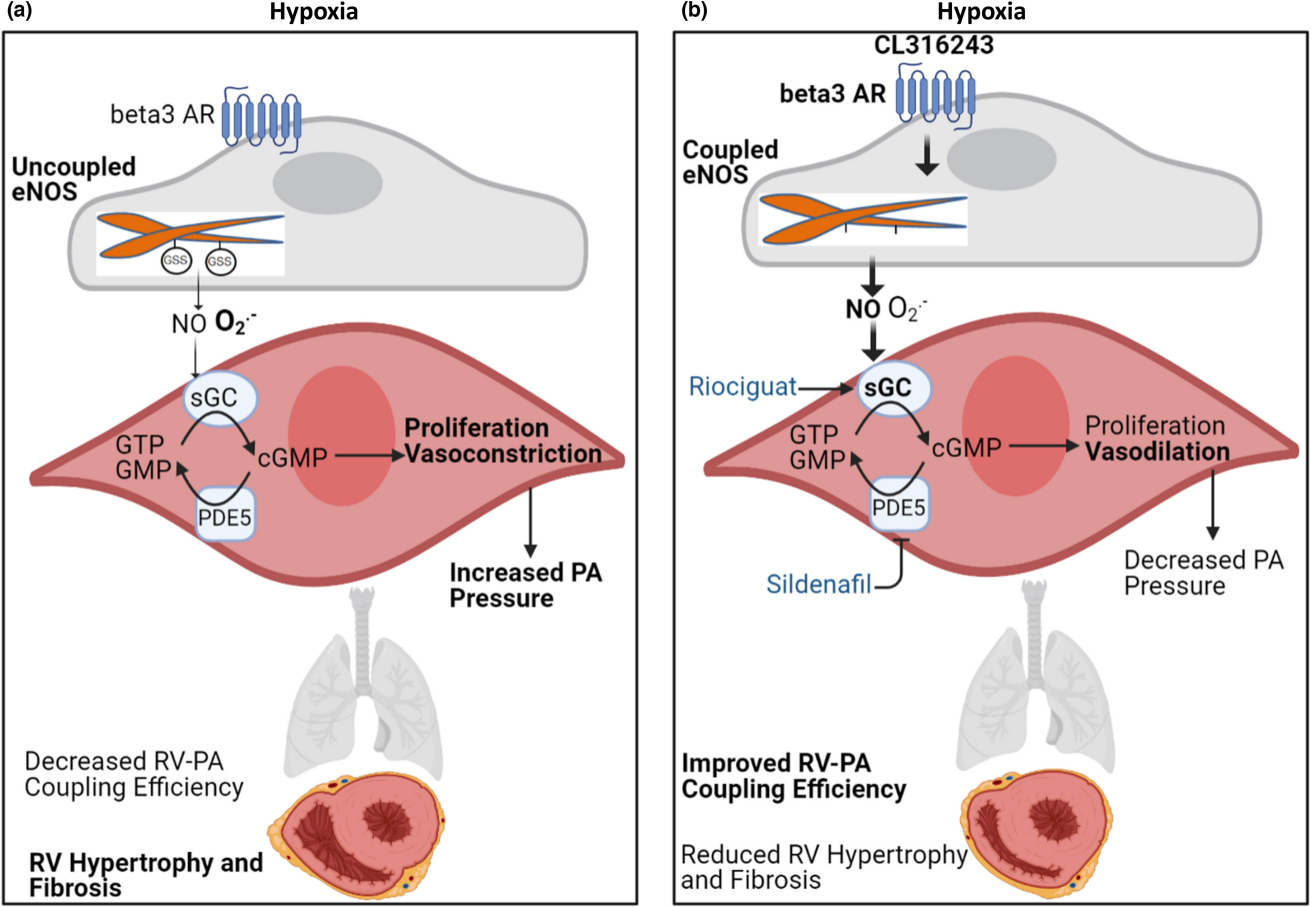
Effects of β3 adrenergic receptor Agonism in experimental pulmonary arterial hypertension. (a) In pulmonary arterial hypertension, endothelial nitric oxide synthase is “uncoupled” via glutathionylation (GSS) of reactive cysteine residues, which leads to the decreased generation of nitric oxide (NO; not shown in this manuscript) and increased generation of $O_{2}^{\cdot -}$. These changes lead to impaired downstream NO‐dependent signaling in smooth muscle cells, resulting in increased vascular proliferation and vasoconstriction, and increased pulmonary arterial (PA) pressure. Consequently, the right ventricular (RV)–PA coupling efficiency is impaired, and RV becomes hypertrophied and fibrosed. (b) Stimulation of β3 adrenergic receptors (β3 ARs) by CL316243 leads to a decrease in the glutathionylation‐mediated uncoupling of eNOS, decreased $O_{2}^{\cdot -}$, and increased NO‐dependent signaling in smooth muscle cells, which leads to decreased proliferation and increased vasodilation, hence a decrease in PA pressure. Sildenafil and riociguat target downstream cellular effectors in NO signaling. The reduction in RV afterload results in improved RV–PA coupling efficiency and reverse remodeling of RV (reduced hypertrophy, and with CL316243, reduced fibrosis). cGMP, cyclic guanosine monophosphate; GMP, guanosine monophosphate; GTP, guanosine triphosphate; PDE5, phosphodiesterase type 5; sGC, soluble guanylyl cyclase.

In the present study, we demonstrate that: (1) chronic exposure to hypoxia (Hx model) or hypoxia with SU5416 (SUHx model) resulted in severe PAH, RV hypertrophy, reduced RV ejection fraction and cardiac output, and muscularization of pulmonary microvasculature, which recapitulate the RV–PA findings in patients with severe PAH; (2) CL316243, a highly selective agonist of β3AR, reduced RV systolic pressure, and hypertrophy to a similar degree to riociguat and sildenafil; (3) CL316243 reduced RV afterload and improved RV–PA coupling efficiency and also improved RV ejection fraction, with the effects generally occurring to a similar magnitude to riociguat and sildenafil; (4) CL316243, akin to sildenafil and riociguat, decreased proliferation of α‐SMA‐positive cells and attenuated pulmonary vascular remodeling; (5) CL316243 decreased oxidative stress and glutathionylation of eNOS and led to upregulation of sGCα1 expression; it thus potentiated the NO‐dependent signaling in the lungs of hypoxic mice. These findings are summarized in the schematic Figure 10 (Visual Abstract).

The β3 ARs are expressed in several human tissues such as the myocardium, endothelial cells, retina, myometrium, adipose tissue, gallbladder, brain, and urinary bladder (Michel et al., 2020). In cardiac myocytes, β3 AR stimulation protects against the deleterious effects of chronic adrenergic stimulation, particularly against hypertrophic remodeling and myocardial fibrosis, through the reduction in oxidative stress, which leads to reduced release of paracrine profibrotic agents (Michel et al., 2020). The reduction in the oxidative stress in the diseased myocardium is mediated via decreased activation of NADPH oxidase (Bundgaard et al., 2010), recoupling of eNOS (Karimi Galougahi et al., 2015), and through a protein kinase G/cGMP/neuronal NOS‐dependent pathway (Hermida et al., 2018). In ischemia–reperfusion, β3 AR stimulation results in NOS‐dependent inhibition of mitochondrial permeability transition pore opening, a trigger of a large increase in oxidative stress and apoptosis, during reperfusion (Garcia‐Prieto et al., 2014). This mechanism may be responsible for the improved left ventricular contractile function and reduced infarct size by β3 AR stimulation in ischemia–reperfusion injury (Garcia‐Prieto et al., 2014). Moreover, β3 ARs expressed in endothelium induce vasorelaxation in the aorta of diabetic rodents (Karimi Galougahi, Liu, et al., 2016). In retinal vessels, β3 ARs mediate antiangiogenesis, with effects on cell proliferation and migration mediated via a signaling cascade that involves mitogen‐activated protein kinase pathway components (Mori et al., 2010).

Clinical application of RV pressure–volume loop analysis, often with methodologic approximations, has shown that lower values of *E*_es_/*E*_a_ (a quantitative measure of RV‐PA coupling) predict mortality in PAH, while increased RV–PA coupling efficiency on PAH‐specific treatment is associated with improved survival (Tabima et al., 2017; Vonk Noordegraaf et al., 2017, 2019). *E*_es_/*E*_a_ ratio markedly decreased in both models of PAH, compared with the normoxic control, which is consistent with maladaptation and RV failure after prolonged exposure (>28 days) to increased afterload in experimental PAH (Wang et al., 2013). CL316243, riociguat, or sildenafil reduced *E*_a_, a composite of PA resistance (static component measured by tPVR) and compliance (dynamic component, not measured in the present study; Tabima et al., 2017). The decrease in *E*_a_ was at least in part due to reduced tPVR, a consequence of the decreased muscularization of the pulmonary microvasculature by treatments.

The RV–PA uncoupling was partially corrected by CL316243, riociguat, or sildenafil, in the SUHx model, as well as by CL316243 or sildenafil in the Hx model. Since the RV energetic input (O_2_ consumption linearly correlating with PVA) and output (external work measured by stroke work) were both reduced by therapies without a change in their ratio (i.e., RV mechanical efficiency), the improved *E*_es_/*E*_a_ ratio was likely due to homeometric adaptation to the reduced afterload without direct inotropic effects on the RV (Liu et al., 2014). We excluded a contribution by LV to the improved RV pump function (Vonk Noordegraaf et al., 2017) by echocardiography, which revealed no changes in the LV systolic function in the hypoxic mice treated with active therapies compared with hypoxic controls.

RV fibrosis is an independent risk factor for poor prognosis in patients with PAH (Freed et al., 2012). Interestingly, only CL316243 reduced RV fibrosis in the Hx model. This finding is akin to the reduction in LV fibrosis, induced by mechanical and neurohormonal stress, by β3 AR activation via a decrease in the paracrine release of profibrotic agents (Hermida et al., 2018). We speculate that the reduction in RV fibrosis in the present study might be a consequence of β3 AR activation in the RV; a postulate that warrants examination in future studies. Additionally, the decrease in the RV afterload may have also contributed to the reduction in the RV fibrosis. Reduction in the RV hypertrophy was reflected in the decrease in Eed, a measure of the RV stiffness, but not in τ (the time constant for RV relaxation) or chamber compliance. This observation likely reflects the detection limits of the conductance‐based pressure–volume technology in the assessment of the relatively subtle functional diastolic changes in the smaller chamber of the RV compared with LV in mice.

The increase in expression of eNOS in the lungs of hypoxic mice is consistent with previous studies reporting increased expression of eNOS in experimental PAH and in the plexiform lesions of patients with PAH.(Berger et al., 2001). However, eNOS expression is not synonymous with NO generation. Indeed, we detected increased levels of eNOS glutathionylation in the lungs of hypoxic mice, which is a reversible oxidative modification of the reactive cysteine residues in the reductive domain of eNOS that leads to the loss of NO generation by eNOS and instead generation of $O_{2}^{\cdot -}$ (Chen et al., 2010). Compared with other mechanisms for eNOS uncoupling, such as eNOS monomerization and tetrahydrobiopterin depletion, glutathionylation is reversible and regulated via receptor‐coupled cell signaling (Chen et al., 2010; Karimi Galougahi, Liu, et al., 2016), thus it may be more pathophysiologically relevant in regulation of eNOS function.

Although all treatments resulted in decreased levels of $O_{2}^{\cdot -}$, CL316243 had a statistically significant effect on eNOS glutathionylation among the treatments used, although there was significant variability in our coimmunoprecipitation data. Nevertheless, the effect of CL316243 in reversing eNOS glutathionylation in the present study is consistent with our previous data showing a similar effect in the systemic vessels (Karimi Galougahi, Liu, et al., 2016) and heart (Karimi Galougahi et al., 2015) of diabetic rodents, and in isolated endothelial cells in vitro (Karimi Galougahi, Liu, et al., 2016). Moreover, this finding is supported by increased eNOS glutathionylation in the heart of mice with genetic knockout of β3 ARs (Karimi Galougahi et al., 2015). This effect may point to the spatial colocalization of β3 ARs with eNOS‐sGC‐cGMP (Michel et al., 2020) and membrane sources of reactive oxygen species such as NADPH oxidase (Karimi Galougahi, Liu, et al., 2016) in the caveolae‐rich membrane rafts. These membrane rafts provide a structural basis for microdomain‐specific redox signaling. We have previously shown that CL316243 results in decreased NADPH oxidase‐mediated $O_{2}^{\cdot -}$ generation and enhancement in colocalization of the deglutathionylation enzyme glutaredoxin‐1 with eNOS and other target proteins in the membrane rafts in endothelial cells (Karimi Galougahi, Liu, et al., 2016) and cardiac myocytes (Karimi Galougahi et al., 2015). These mechanisms may also be responsible for the effects of CL316243 on redox stress and eNOS glutathionylation in the lungs of hypoxic mice.

These micordomain‐specific effects of β3 ARs on eNOS glutathionylation in the endothelial cells are distinct compared with the redox effects of sildenafil and riociguat. NO generated in the endothelial cells diffuses into the cytoplasm of underlying smooth muscle cells and activates sGC, increasing the generation of the second messenger cGMP and subsequently cGMP‐dependent protein kinase G (Humbert et al., 2019). Activation of protein kinase G stimulates Ca^2+^‐activated potassium channels, which leads to membrane hyperpolarization and vasodilation. In the lung, cGMP is metabolized by PDE5 (Hansen et al., 2016). PDE5 inhibitors (e.g., sildenafil), and riociguat, which stimulates sGC and thereby cGMP availability, are approved for the treatment of PAH (Humbert et al., 2019). These drugs improve pulmonary vascular hemodynamics as shown in the present study. Improvement in the pulmonary vascular hemodynamics mediated by the direct vasodilatory effect of riociguat and sildenafil can decrease the biomechanical stimuli that drive pathologic activation of several sources of $O_{2}^{\cdot -}$ in smooth muscle cells (Karimi Galougahi, Ashley, et al., 2016), thus reducing the overall $O_{2}^{\cdot -}$ levels, without impacting eNOS glutathionylation.

The heterodimeric α/β sGC is the downstream target of NO in the canonical NO‐dependent signaling (Lang et al., 2012). In accordance with a previous report (Lang et al., 2012), we detected a decrease in the expression of the sGCα1 but not the β1 subunit in the lungs of hypoxic mice compared with the normoxic controls. Consistent with the effect of CL316243 to reverse a mechanism for uncoupling of eNOS, expression of sGCα1 was increased by CL316243 to a similar degree to the direct sGC stimulator riociguat, which has previously been shown to increase sGCα1 expression (Lang et al., 2012). These data suggest the potentiation of the downstream NO signaling by CL316243.

The effect of CL316243 on RV–PA hemodynamics and pulmonary vascular remodeling was similar in magnitude to sildenafil and riociguat. Nevertheless, reversing an oxidative mechanism of eNOS uncoupling appeared to be unique to CL316243. PDE5 inhibitors (sildenafil and tadalafil) competitively bind to the catalytic domain of PDE5 and prevent cGMP binding and hydrolysis (Klinger & Kadowitz, 2017), while riociguat binds to the reduced (heme‐bound) form of sGC to activate sGC via two different mechanisms: stabilizing the NO–sGC complex in the presence of NO (Klinger & Kadowitz, 2017; Lang et al., 2012) and activating sGC directly in the absence of NO by binding to a separate regulatory site (Stasch et al., 2001). The efficacy of PDE5 inhibitors is dependent on the availability of cGMP, which in turn is predominantly dependent on the bioavailability of NO (Klinger & Kadowitz, 2017). Since NO level is progressively decreased as PAH worsens, the efficacy of PDE5 inhibitors as monotherapy or the NO‐dependent activity of sGC stimulators used as monotherapy may decrease (Klinger & Kadowitz, 2017). The putative effect of β3 AR stimulation to reverse a mechanism for uncoupling of eNOS upstream of sGC‐cGMP signaling may provide a theoretical rationale for the use of β3 AR agonists as an alternative agent if sildenafil or riociguat monotherapy is not effective; a hypothesis that warrants testing in clinical trials. Additionally, the resistance of β3 ARs to agonist‐induced desensitization in response to sustained stimulation (Michel et al., 2020) may suggest a low likelihood of tachyphylaxis to β3 AR agonists in PAH. In contrast to PDE5 inhibitors and sGC stimulators that infrequently cause systemic hypotension (i.e., in 8% (Rubin et al., 2011) and 10% (Rubin et al., 2015) of patients, respectively; Thenappan et al., 2018), β3 AR agonists do not decrease the systemic arterial pressure (Michel et al., 2020). Akin to combination therapy with sildenafil and riociguat, shown to excessively potentiate the NO pathway leading to significant systemic hypotension without additional benefits in PAH (Galie et al., 2015), combining β3 AR agonists upstream of sGC‐PDE5 with agents targeting these downstream molecules may cause excessive potentiation of NO signaling, and should be avoided. Thus, the safety and efficacy of β3 AR agonists as an alternative treatment, and not combination therapy, in patients in whom monotherapy with PDE5 inhibitors or sGC stimulators is either not effective or poorly tolerated warrants testing in clinical trials.

Hemodynamic effects of β3 AR agonism in a porcine model of pulmonary venous banding, replicating pulmonary hypertension secondary to left heart failure (group 2 pulmonary hypertension) have been reported (Garcia‐Alvarez et al., 2016). Nonetheless, both the pathobiology and hemodynamic profile of PAH are fundamentally different compared with group 2 pulmonary hypertension. Moreover, PAH‐specific therapies are ineffective or even harmful in heart failure with reduced ejection fraction (Rosenkranz et al., 2016). In this regard, mirabegron, a selective β3 AR agonist that is approved for use in overactive bladder syndrome, did not impact the LV ejection fraction in a small randomized study in patients with heart failure and reduced ejection fraction (Bundgaard et al., 2017). Moreover, the beneficial effect of nebivolol in a rat model of PAH has been attributed to β3 AR agonism (Perros et al., 2015), nevertheless, pharmacodynamic studies have established nebivolol as a very selective β1 AR ligand with no agonist efficacy for β3 ARs (Baker, 2010). The present study is the first to report beneficial hemodynamic effects of a selective β3 AR agonist in experimental PAH, suggesting β3 AR agonists may be effective for the treatment of this distinct group of patients with pulmonary hypertension.

### 4.1 Limitations

First, we used two mouse models of PAH, which have known limitations and pathophysiological differences from PAH in humans (Vitali et al., 2014). Despite these differences, pathological and biochemical findings in the Hx and SUHx models are acceptable experimental approximations of pathobiological features in clinical PAH. While pulmonary vascular remodeling in the Hx model was relatively mild, SHx recapitulated a relatively more severe PAH phenotype in the present study and has been previously shown to induce the plexiform vascular lesions that are observed in human PAH (Vitali et al., 2014). Nevertheless, of the two models, there is more data on the contribution of the Hx model to the development of therapeutic agents for PAH that are in clinical use (Vitali et al., 2014). Second, we used the SHx model, a more severe model compared with the Hx model, to assess the hemodynamic effects of CL316243, and have not fully characterized the morphologic and molecular effects of the study drug in this model. Third, we did not test the impact of the genetic deletion of β3 AR on the observed effects of CL316243. Nevertheless, CL316243 is approximately 130‐fold more selective for β3 AR over β1 AR and >10‐fold more selective over β2 AR (Baker, 2005). Moreover, we have previously shown that the receptor‐coupled, redox‐mediated signaling by CL316243 is completely blocked by L748337, a highly selective competitive antagonist of β3 AR (Baker, 2005), whereas nadolol, which blocks the β1/β2 ARs (Baker, 2005), had no impact on the effects of CL316243 (Bundgaard et al., 2010). Fourth, while combining β3 AR agonists with agents targeting sGC‐PDE5 may cause excessive potentiation of NO signaling and not be clinically useful, β3 AR agonists could be combined with endothelin‐receptor antagonists. This combination therapy could be tested in future basic experimental studies, and we are currently testing this combination in a clinical trial. Last, we used male animals only to avoid the potential confounding impact of gender on outcomes.

In conclusion, despite advances in the medical treatment of PAH, mortality due to this condition remains high, and novel therapeutic approaches are required. Findings of the present study in experimental PAH using the highly selective β3 AR agonist CL316243 in rodents have provided the rationale for Beta3 Adrenergic Agonists in Pulmonary Hypertension (BEAT‐PH) trial (ACTRN12620001349932, http://www.anzctr.org.au/). The BEAT‐PH trial is a proof‐of‐concept, acute hemodynamic study of mirabegron—the only highly selective β3 AR agonist currently in use in humans (approved for overactive bladder syndrome)—which our group is currently conducting in precapillary pulmonary hypertension.

## AUTHOR CONTRIBUTIONS

Keyvan Karimi Galougahi conceived the idea, designed and performed the experiments, analyzed and interpreted the data, and drafted the manuscript; Yunjia Zhang and Vivian Kienzle designed and performed the experiments and analyzed and interpreted the data; Chia‐Chi Liu and Lake‐Ee Quek designed the experiments, analyzed and interpreted the data; Sanjay Patel, Edmund Lau, Rachael L. Cordina, and Gemma A. Figtree contributed to the design and analysis and interpretation of data; and David S. Celermajer contributed to the conception, design of the study, and analysis and interpretation of the data. All authors read and edited the manuscript and approved the final version for submission.

## FUNDING INFORMATION

This work was funded by a New South Wales Health Early‐to‐Mid Career Fellowship (H18/31086), Sydney, Australia (awarded to KKG).

## CONFLICT OF INTEREST

The authors declare that they have no conflict of interest relevant to the content of this manuscript.
